# Mortality of populations potentially exposed to ionising radiation, 1953–2010, in the closed city of Ozyorsk, Southern Urals: a descriptive study

**DOI:** 10.1186/s12940-015-0078-8

**Published:** 2015-11-27

**Authors:** Isabelle Deltour, Fyodor Tretyakov, Yulia Tsareva, Tamara V. Azizova, Joachim Schüz

**Affiliations:** Section of Environment and Radiation, International Agency for Research on Cancer (IARC), 150 cours Albert Thomas, F-69372 Lyon, France; Clinical Department Southern Urals Biophysics Institute (SUBI), Ozyorsk, Russian Federation; Epidemiology Laboratory, Southern Urals Biophysics Institute (SUBI), Ozyorsk, Russian Federation

**Keywords:** Nuclear workers, Cause specific mortality, All cause mortality, Ozyorsk, Southern Urals, Russian Federation, Soviet Union, Ionising radiation, Plutonium, Cancer

## Abstract

**Background:**

The city of Ozyorsk (Southern Urals) was created as a secret city in 1945 and is a closed city until today. It housed workers of the earliest and one of the country’s largest nuclear facilities. Workers of the nuclear reactors, radiochemical or reprocessing plants were exposed to high levels of ionising radiation in the early years of operation and possibly further exposed from inhalation of plutonium aerosols.

**Methods:**

The cause-of-death registry of Ozyorsk received paper copies of original death certificates of all deaths of residents of the city. Data were analysed for recent mortality rates (1998–2010) and time trends in age-standardised mortality rates between 1953 and 2010 of main groups of causes of deaths, in particular cancer.

**Results:**

Comparing workers of the three main plant types with the remainder of the Ozyorsk residents, and with national figures, all-cause mortality rates were lowest among workers, with ratios compared to national figures of 0.65 (men) and 0.56 (women), and compared to the other residents of 0.77 (men) and of 0.74 (women). For cancer overall, the differences were smaller in men (ratio between workers and national figures of 0.86) and there were no differences in women (ratio of 1.00), but ratios differed by cancer type. Most cancer deaths were however least common in the workers, including leukaemia. Over the last 60 years, all-cause mortality has gradually increased among men in all three groups but was stable among women, whereas cancer death rates have slightly declined in both sexes.

**Conclusions:**

Healthy worker effect, relatively better living conditions in Ozyorsk and healthier lifestyles may explain the lower mortality rates in Ozyorsk. Overall mortality time trends in Ozyorsk were similar to the entire country. No apparent radiation-related effects were seen in this population-level analysis, but the radiation-related risks can be better addressed in individual-level studies.

**Electronic supplementary material:**

The online version of this article (doi:10.1186/s12940-015-0078-8) contains supplementary material, which is available to authorized users.

## Background

The first Soviet production facility of nuclear weapons, Mayak Production Association (Mayak PA) started operation in the Southern Urals in 1948 [1, 2]. The main plants of Mayak PA consist of several nuclear reactors, a radiochemical plant, and a reprocessing plant. Since commissioning, the workforce exposure to ionising radiation has been continuously monitored. Practices of workers’ radioprotection evolved considerably over the years of operation of the facility. In the early years of operation (1948–1953), workers were exposed to very high doses. The workforce’s mean annual doses were around 200 mGy or higher until 1953 for external exposures, with gradual decreases afterwards in the 3 main plant types. In the last 20 years, the 3-plants workers’ mean annual dose of external exposures did not exceed 5 mGy. Some individuals accumulated doses of external exposures of up to 10 Gy during the prolonged period. In addition, some workers were at risk of inhaling plutonium aerosols, with the highest rates of plutonium intake in years 1949–1958 when individual devices for protection of respiratory organs were not available. About a quarter of the workforce were women.

All workers employed at Mayak PA lived in a closed city 5 to 10 km away from the facility, called first Chelyabinsk-65, then, from 1966 onwards, Chelyabinsk- 40 and Ozyorsk since 1994. The city was created in 1945 for the purpose of housing current and former Mayak employees and their families; in 2013, the still closed city had about 81 000 inhabitants. This city was formerly secret, and population movements in and out of town were recorded and restricted. During the 1950’s, 34 % of those working there at the time left the city, partly due to migration of some specialists to newly opened similar plants in the area, partly due to removal of some of the workforce in relation to the severe working conditions at Mayak PA at the time, and partly due to less restricted conditions for departure from the city. In contrast, since the 1970’s, the worker’s population was stable (0-1 % migration). Social and economic conditions of residence in Ozyorsk (salary, apartment provided etc.) were much better than in other places of residence.

The cause-of-death register of Ozyorsk was established at the Southern Urals Biophysics Institute (SUBI) in 1988 [3]. Based on copies of the original paper death certificates, it included deaths from all causes in the city since 1948. The completeness of data in the register is virtually 100 %. Additionally, quality assurance checks of the cause-of-death information have been performed for Mayak workers in the framework of previous epidemiological studies [4, 5] in which it was found that 80 %, 25 %, and 50 % of death certificates were confirmed by autopsy before 1990, during 1990 –2000, and after 2000, respectively. Malignant neoplasms were found to be histologically verified for 80 % of all cancer deaths.

The objective of this study was to describe the mortality of the population of Ozyorsk and cancer mortality in particular. We compared the causes of deaths of workers of the 3 main plant types (nuclear reactors, radiochemical plant, reprocessing plant) with those of the remainder of the Ozyorsk population and with the national figures of the Russian Federation in the most recent time period (1998–2010). We also described long-term time trends of the mortality rates in these populations (1973–2010).

## Methods

### Data

Numbers of deaths in people aged 18 to 74 years between 1953 and 2010 were obtained from the Ozyorsk cause-of-death register [3]. They were subdivided into 2 population groups covering the entire closed city: the people ever employed at one of the 3 main Mayak plant types (reactors, radiochemical and reprocessing plants), and the remainder of the Ozyorsk population. The latter also included the other Mayak PA workers not working at any of the 3 plant types. The two groups will be called the “3-plants workers” and the “other residents” for simplicity. For the 3 plants workers, size of the population at risk by 5-years age groups for each 5-year calendar time periods was computed from the Mayak PA employment records. For the other residents, the size was computed from the lists of electors of city and state authority elections containing all the city citizens aged 18 years or older for the period before 1958, and later, from the census information in years 1959, 1970, 1979, 1989, 2002 and 2010 and the local statistics bureau for population information, from which the 3-plant workers population was subtracted. Only Ozyorsk residents contributed person-years at risk until death, migration or 2010, whichever came first. People working at Mayak PA contributed person-years to the 3-plants workers population from the time when they started working in one of the 3 plant types. For comparison, deaths rates in the Soviet Union until 1991 and in the Russian Federation afterwards were obtained from the WHO database [6] for the longest possible period of time.

The cause-of-death register of Ozyorsk codes diagnoses according to the Russian version of the ninth revision of the International Classification of Diseases (ICD-9). The register received copies of the original paper death certificate and coding is done manually by registry staff. Mortality from all causes of deaths (ICD-9 codes 001–999) was analysed. We also investigated deaths from all malignant neoplasms (codes 140–209; called “cancers” in this manuscript), and specifically deaths from cancers of lip, oral cavity and pharynx (codes 140–149), of digestive organs and peritoneum (codes 150–159), of the respiratory and intrathoracic organs (codes 160–165), of the bone, connective tissue, skin and breast (codes 170–175), of the genitourinary organs (codes 179–189), as well as other and unspecified malignant cancers (codes 189–199), and malignancies of the lymphatic and hematopoietic tissue (codes 200–208). Deaths from cancers of selected specific organs were also considered: stomach (code 151), rectum (code 154), liver (code 155), pancreas (code 157), larynx (code 161), lung (code 162), pleura (code 163), female breast (code 174), multiple myelomas (code 203), and leukemia (codes 204–208). In addition, deaths from infections (codes 001–139), from diseases of the circulatory system (codes 390–459) with subgroups ischemic heart disease (codes 410–414) and cerebrovascular diseases (codes 430–438), from respiratory system diseases (codes 460–519), from digestive system diseases (codes 520–579), from disease of the genitourinary system (codes 580–629), from injury and poisoning (codes 800–999), and from other causes of deaths (codes 210–389 and 630–799) were also investigated separately, whenever possible.

### Statistical analysis of observed mortality rates

Age standardised mortality rates (ASR) of the various groupings of causes of death per 100,000 person-years were calculated separately for men and women. The European standard population, truncated to 18–74 years, was used for age standardisation. Ninety-five percent confidence intervals were computed using the gamma approximation [7]. For the most recent time period, 1998–2010, the standardised rate ratios (SRR) were the ratios of ASR in 3-plants workers compared to other residents and to the national figures, and in the other residents compared to the national figures. Confidence intervals of the SRR did not account for the variability of the national figures due to their large population size. Figures show the mortality rates of main causes of deaths during the period 1953 to 2010 for the age group 18 to 49 years, and for 18 to 74 years. Long-term time trends were described by average annual percent changes in ASR for the period 1973 to 2010 in 18–74 years old (Joinpoint Regression Program, version 3.4.3 – April 2010; Statistical Research and Applications Branch, National Cancer Institute). The 3-plants workers population above 50 years old was too small for analysis for the period 1953 to 1972, and for some sex and cause-of-death combinations. When numbers were smaller than 250 person-years in a specific age and time period group, this age group was merged with the neighbouring age group to enlarge it. Truncated standard population weights were then adapted accordingly.

## Results

In the recent period 1998–2010, a total of 8677 deaths was observed among 18 to 74 years old in Ozyorsk in 833,968 person-years (Tables 1, 2). All cause ASR were about three times higher in men than women in all three population groups; 3 times in the 3 plant workers (men: 1332 (95 % CI 1273, 1394) and women: 441 (95 % CI 396, 535)), 2.9 times in the other residents (men: 1733 (95 % CI 1676, 1793) and women: 597 (95 % CI 574, 620)), and 2.8 times in the national figures (men: 2009, women 730).

**Table 1:** Male adult mortality rates in Mayak 3-plants workers, other Ozyorsk residents and in Russian Federation, 1998–2010

		3-Plants workers			Other Ozyorsk residents			Russian federation	3-Plants workers to other residents		Other residents to Russia		3-Plants workers to Russia	
Cause-of-death	ICD-9 code	N	ASR	(95 % CI)	N	ASR	(95 % CI)	ASR	SRR	(95 % CI)	SRR	(95 % CI)	SRR	(95 % CI)
All	001–999	2043	1332	(1273, 1394)	3614	1733	(1676, 1793)	2009	0.77	(0.73, 0.81)	0.86	(0.83, 0.89)	0.66	(0.63, 0.69)
All cancers	140–209^+^	434	262	(237, 290)	547	295	(270, 321)	304	0.89	(0.78, 1.01)	0.97	(0.89, 1.05)	0.86	(0.78, 0.95)
Lip, oral cavity, pharynx c.	140–149	14	8	(4, 17)	37	19	(13, 27)	15	0.43	(0.23, 0.80)	1.26	(0.91, 1.74)	0.54	(0.32, 0.92)
Digestive organs and peritoneum c.	150–159	163	99	(84, 117)	213	117	(101, 134)	NA	0.85	(0.69, 1.04)	NA		NA	
*Stomach cancer*	*151*	*55*	*34*	*(25, 46)*	*64*	*35*	*(27, 45)*	*44*	*0.96*	*(0.67, 1.38)*	*0.80*	*(0.62, 1.02)*	*0.77*	*(0.59, 1.00)*
*Rectum cancer*	*154*	33	20	(13, 30)	43	24	(17, 32)	13^a^	*0.83*	*(0.52, 1.31)*	*1.77*	*(1.31, 2.40)*	*1.46*	*(1.04, 2.06)*
*Liver cancer*	*155*	3	2	(0, 9)	9	5	(2, 9)	9^b^	*0.35*	*(0.09, 1.29)*	*0.54*	*(0.28, 1.04)*	*0.19*	*(0.06, 0.58)*
*Pancreas cancer*	*157*	28	17	(11, 28)	33	18	(12, 26)	14^b^	*0.96*	*(0.58, 1.60)*	*1.28*	*(0.91, 1.81)*	*1.23*	*(0.84, 1.79)*
Respiratory and intrathoracic c.	160–165	143	82	(69, 99)	157	87	(74, 102)	NA	0.95	(0.76, 1.19)	NA		NA	
*Larynx cancer*	*161*	*15*	*9*	*(5, 18)*	*25*	*13*	*(8, 20)*	*10* ^b^	*0.68*	*(0.35, 1.29)*	*1.26*	*(0.85, 1.88)*	*0.85*	*(0.51, 1.43)*
*Lung cancer*	*162*	*125*	*72*	*(60, 88)*	*126*	*71*	*(59, 84)*	*94*	*1.02*	*(0.79, 1.30)*	*0.75*	*(0.63, 0.90)*	*0.77*	*(0.64, 0.91)*
Bone, connective tissues, skin c.	170–175	13	9	(5, 18)	15	7	(4, 12)	NA	1.25	(0.59, 2.67)	NA		NA	
Genitourinary organs c.	179–189	49	29	(21, 41)	56	31	(23, 41)	NA	0.93	(0.63, 1.37)	NA		NA	
Other and unspecified c.	190–199	33	22	(15, 34)	31	14	(10, 21)	NA	1.53	(0.92, 2.55)	NA		NA	
Haematological malignancies	200–208	19	13	(7, 23)	38	19	(14, 27)	14 ^a^	0.65	(0.36, 1.18)	1.37	(0.99, 1.90)	0.89	(0.54, 1.47)
*Multiple myeloma*	*203*	*5*	*3*	*(1, 10)*	*8*	*4*	*(2, 8)*	*1* ^b^	*0.66*	*(0.21, 2.03)*	*2.82*	*(1.39, 5.69)*	*1.86*	*(0.77, 4.47)*
*Leukemia*	*204*–*208*	*6*	*4*	*(1, 11)*	*19*	*9*	*(6, 15)*	*7*	*0.38*	*(0.15, 0.96)*	*1.38*	*(0.87, 2.20)*	*0.52*	*(0.23, 1.17)*
Infectious and parasitic diseases	001–139	15	10	(6, 19)	42	18	(13, 24)	55	0.56	(0.31, 1.03)	0.32	(0.24, 0.44)	0.18	(0.11, 0.30)
Circulatory system diseases	390–459	882	543	(507, 582)	1374	731	(692, 771)	905	0.74	(0.68, 0.81)	0.81	(0.77, 0.85)	0.60	(0.56, 0.64)
*Ischemic heart disease*	*410*–*414*	*555*	*343*	*(315, 375)*	*841*	*443*	*(413, 474)*	*488*	*0.77*	*(0.69, 0.86)*	*0.91*	*(0.85, 0.97)*	*0.70*	*(0.65, 0.76)*
*Cerebrovascular disease*	*430*–*438*	*244*	*146*	*(128, 167)*	*378*	*211*	*(190, 234)*	*251*	*0.69*	*(0.59, 0.81)*	*0.84*	*(0.76, 0.93)*	*0.58*	*(0.51, 0.66)*
Respiratory system diseases	460–519	50	31	(23, 43)	100	50	(41, 62)	113	0.62	(0.44, 0.87)	0.44	(0.36, 0.54)	0.27	(0.21, 0.36)
Digestive system diseases	520–579	127	83	(69, 100)	180	87	(74, 101)	91	0.95	(0.75, 1.20)	0.95	(0.82, 1.11)	0.91	(0.76, 1.08)
Genitourinary system diseases	580–629	13	8	(4, 16)	26	12	(8, 19)	11	0.62	(0.32, 1.22)	1.08	(0.73, 1.61)	0.67	(0.39, 1.17)
Other causes of death	210–389, 630–799	230	161	(140, 185)	488	214	(195, 235)	108	0.75	(0.64, 0.88)	1.99	(1.81, 2.18)	1.49	(1.30, 1.70)
Injury and poisoning	800–999	292	236	(208, 267)	857	326	(304, 350)	423	0.72	(0.63, 0.83)	0.77	(0.72, 0.83)	0.56	(0.49, 0.63)

Legend: All cause and cause-specific age standardized mortality rates in men per 100 000 male population aged 18–74 years in the workers of the 3 main plant types of Mayak, in the other Ozyorsk residents, and in Russian Federation, in years 1998–2010, and standardised rate ratios. Abbreviations: ICD-9 International Classification of Diseases revision 9, N observed number of deaths, ASR Age standardized rate, CI confidence interval, SRR standardized rate ratio, c. cancers. ^a^only available for year 1998. ^b^only available between 1999 and 2010. ^+^No deaths from code 209 observed

**Table 2:** Female adult mortality rates in 3-plants Mayak workers, other Ozyorsk residents and in Russian Federation, 1998–2010

		3-Plants workers			Other Ozyorsk residents			Russian federation	3-Plants workers to other residents		Other residents to Russia		3-Plants workers to Russia	
Cause-of-death	ICD-9 code	N	ASR	(95 % CI)	N	ASR	(95 % CI)	ASR	SRR	(95 % CI)	SRR	(95 % CI)	SRR	(95 % CI)
All	001–999	419	441	(396, 535)	2601	597	(574, 620)	730	0.74	(0.66, 0.83)	0.82	(0.79, 0.85)	0.60	(0.54, 0.67)
All cancers	140–209^+^	131	149	(122, 232)	672	155	(144, 168)	148	0.96	(0.78, 1.17)	1.05	(0.97, 1.13)	1.01	(0.83, 1.22)
Lip, oral cavity, pharynx c.	140–149	1	1	(0, 90)	12	3	(1, 5)	2	0.25	(0.03, 1.90)	1.57	(0.89, 2.78)	0.39	(0.05, 2.76)
Digestive organs and peritoneum c.	150–159	46	49	(35, 131)	234	53	(46, 60)	NA	0.92	(0.65, 1.29)	NA		NA	
*Stomach cancer*	*151*	*14*	*13*	*(7, 99)*	*68*	*16*	*(12, 20)*	*17*	*0.84*	*(0.45, 1.55)*	*0.92*	*(0.73, 1.17)*	*0.77*	*(0.44, 1.36)*
*Rectum cancer*	*154*	*9*	*11*	*(5, 97)*	*34*	*8*	*(5, 11)*	*8* ^a^	*1.44*	*(0.66, 3.16)*	*0.91*	*(0.65, 1.27)*	*1.31*	*(0.64, 2.65)*
*Liver cancer*	*155*	*4*	*4*	*(1, 92)*	*9*	*2*	*(1, 4)*	*4* ^b^	*1.92*	*(0.57, 6.46)*	*0.54*	*(0.28, 1.05)*	*1.04*	*(0.38, 2.88)*
*Pancreas cancer*	*157*	*4*	*4*	*(1, 92)*	*40*	*9*	*(7, 13)*	*7* ^b^	*0.38*	*(0.13, 1.14)*	*1.37*	*(1.00, 1.87)*	*0.53*	*(0.19, 1.48)*
Respiratory and intrathoracic c.	160–165	12	13	(7, 99)	37	8	(6, 12)	NA	1.58	(0.80, 3.13)	NA		NA	
*Larynx cancer*	*161*	*1*	*1*	*(0, 90)*	*1*	*0*	*(0, 2)*	*0* ^b^	*3.67*	*(0.23, 58.7)*	*0.97*	*(0.14, 6.88)*	*3.56*	*(0.50, 25.3)*
*Lung cancer*	*162*	*11*	*12*	*(6, 98)*	*34*	*8*	*(5, 11)*	*9*	*1.61*	*(0.79, 3.28)*	*0.84*	*(0.60, 1.18)*	*1.35*	*(0.72, 2.53)*
Bone, connective tissues, skin, breast c.	170–175	31	37	(24, 121)	161	39	(33, 45)	NA	0.97	(0.63, 1.48)	NA		NA	
*Breast cancer*	*174*	*23*	*25*	*(15, 109)*	*144*	*35*	*(29, 41)*	*29*	*0.72*	*(0.44, 1.16)*	*1.19*	*(1.01, 1.41)*	*0.86*	*(0.54, 1.35)*
Genitourinary organs c.	179–189	28	33	(21, 116)	127	29	(24, 35)	25	1.13	(0.73, 1.74)	1.20	(1.00, 1.43)	1.35	(0.91, 2.01)
Other and unspecified c.	190–199	6	8	(2, 95)	56	13	(10, 17)	NA	0.61	(0.23, 1.59)	NA		NA	
Haematological malignancies	200–208	7	8	(3, 95)	45	10	(8, 14)	9^a^	0.78	(0.32, 1.86)	1.20	(0.89, 1.61)	0.93	(0.41, 2.12)
*Multiple myeloma*	*203*	*4*	*4*	*(1, 92)*	*13*	*3*	*(2, 5)*	*1* ^b^	*1.44*	*(0.41, 4.99)*	*2.34*	*(1.35, 4.04)*	*3.36*	*(1.10, 10.25)*
*Leukemia*	*204*–*208*	*2*	*2*	*(0, 91)*	*15*	*3*	*(2, 6)*	*4*	*0.59*	*(0.12, 2.81)*	*0.78*	*(0.47, 1.30)*	*0.46*	*(0.10, 2.01)*
Infectious and parasitic diseases	001–139	1	1	(0, 90)	17	4	(2, 7)	11	0.15	(0.02, 1.14)	0.39	(0.24, 0.62)	0.06	(0.01, 0.41)
Circulatory system diseases	390–459	201	184	(157, 266)	1106	244	(230, 259)	366	0.75	(0.64, 0.88)	0.67	(0.63, 0.71)	0.50	(0.43, 0.58)
*Ischemic heart disease*	*410*–*414*	*104*	*98*	*(7, 180)*	*493*	*110*	*(101, 121)*	*162*	*0.89*	*(0.71, 1.12)*	*0.68*	*(0.62, 0.74)*	*0.61*	*(0.49, 0.75)*
*Cerebrovascular disease*	*430*–*438*	*70*	*57*	*(44, 138)*	*465*	*100*	*(91, 109)*	*138*	*0.57*	*(0.44, 0.75)*	*0.72*	*(0.66, 0.79)*	*0.41*	*(0.32, 0.53)*
Respiratory system diseases	460–519	8	8	(3, 95)	45	10	(7, 13)	22	0.76	(0.341.69)	0.45	(0.33, 0.60)	0.34	(0.16, 0.72)
Digestive system diseases	520–579	21	26	(15, 110)	161	38	(33, 45)	42	0.68	(0.41, 1.11)	0.92	(0.79, 1.08)	0.63	(0.39, 1.00)
Genitourinary system diseases	580–629	3	3	(1, 91)	44	10	(7, 14)	8	0.25	(0.08, 0.81)	1.34	(0.99, 1.80)	0.33	(0.10, 1.05)
Other causes of death	210–389, 630–799	32	43	(28, 126)	280	68	(60, 76)	43	0.63	(0.42, 0.94)	1.59	(1.41, 1.79)	1.00	(0.68, 1.47)
Injury and poisoning	800–999	22	29	(17, 113)	276	67	(60, 76)	91	0.44	(0.27, 0.71)	0.74	(0.66, 0.83)	0.32	(0.20, 0.51)

Legend: All cause and cause-specific age standardised mortality rates in women per 100 000 female population aged 18–74 years in the workers of the 3 main plant types of Mayak, other Ozyorsk residents, Russian Federation, in years 1998–2010, No death from code 209 observed. Abbreviations: ASR Age standardised rates, CI confidence intervals, c. cancers. ^a ^Only available for year 1998. ^b ^Only available between 1999 and 2010. ^+^No deaths from code 209 observed

All causes ASR was lower among 3-plants workers than among other residents, with a SRR of 0.77 (95 % CI 0.73, 0.81) in men and of 0.74 (95 % CI 0.66, 0.83) in women. The leading cause-of-death was from circulatory system diseases, with the rates being similarly lower than for all causes mortality, namely of 0.74 (95 % CI 0.68, 0.81) in men and of 0.75 (95 % CI 0.64, 0.88) in women.

Cancer was the second most common cause-of-death for both male and female 3-plants workers. Cancer death rates were generally lower among 3 plants workers than among other residents in men (SRR = 0.89 (95 % CI 0.78, 1.01)), while they were about the same among women (SRR = 0.96 (95 % CI 0.78, 1.17)). No single statistically significantly elevated SRR was observed in any specific cancer type for 3 plants workers compared to other residents, but some SRRs were nevertheless increased. Among men, these were cancers of other and unspecified sites (SRR = 1.53 (95 % CI 0.92, 2.55)), and bone, skin and connective tissue cancers (SRR = 1.25 (95 % CI 0.59, 2.67)), while for lung cancers rates were equal (SRR = 1.02 (95 % CI 0.79, 1.30)). Elevated SRRs among female 3-plants workers were seen for liver cancer (SRR = 1.92 (95 % CI 0.57, 6.46)), lung cancers (SRR = 1.61 (95 % CI 0.79, 3.28)), rectum cancers (SRR = 1.44 (95 % CI 0.66, 3.16)), multiple myelomas (SRR = 1.44, (95 % CI 0.41, 4.99))and for genitourinary cancers (SRR = 1.13 (95 % CI 0.73, 1.74)), compared to other female residents (considering only cancers with at least 4 observed deaths). For several cancer sites SRRs of 3-plants workers compared to the other residents were decreased. This was statistically significantly so, in men, for leukemia (SRR = 0.38 (95 % CI 0.15, 0.96) and for lip, oral cavity, and, pharynx cancers (SRR = 0.43 (95 % CI 0.23, 0.80).

Injury and poisoning was the third most common cause-of-death among male workers, while it ranked second among other male residents, resulting in a SRR of 0.72 (95 % CI 0.63, 0.83)). Few women died of injuries and poisoning. ASR from all other groups of causes of deaths were lower among the 3-plants workers compared to the other residents.

Mortality rates in Ozyorsk were generally lower than the national figures of the Russian Federation. The all causes SRR between the 3-plants workers and the national figures was as low as 0.66 (95 % CI 0.63, 0.69) in men and 0.60 (95 % CI 0.54, 0.67) in women. Patterns by main causes of death were similar: SRRs were lowest for infectious and parasitic diseases (SRR = 0.18 (95 % CI 0.11, 0.30) in men, SRR = 0.06 (95 % CI 0.01, 0.41) in women), also very low for respiratory diseases (SRR = 0.27 (95 % CI 0.21, 0.36) in men, 0.34 (95 % CI 0.16 , 0.72) in women), for injuries and poisoning they were low in men (SRR = 0.56 (95 % CI 0.49, 0.63)) and very low in women (SRR = 0.32 (95 % CI 0.20, 0.51)), and also low for circulatory diseases (SRR = 0.60 (95 % CI 0.56, 0.64) in men and SRR = 0.50 (95 % CI 0.43, 0.58)) in women). For cancer on the other hand the SRR was only decreased in men (SRR = 0.86 (95 % CI 0.78, 0.95) but not in women (SRR = 1.01 (95 % CI 0.83, 1.22)). For deaths from other causes, the SRR was high in men (SRR = 1.49 (95 % CI 1.30, 1.70)) while no difference was seen for women (SRR = 1.00).

For some cancer subtypes SRRs were even elevated comparing 3 plants workers with the national figures. For multiple myelomas, the SRR was high in men (SRR = 1.86 (95 % CI 0.77, 4.47)) and very high in women (SRR =3.36 (95 % CI 1.10, 10.25)). For rectal cancers, the SRR was high in men (1.46 (95 % CI 1.04, 2.06)), but showed less difference for women (1.31 (95 % CI 0.64, 2.65).

**Fig. 1:**
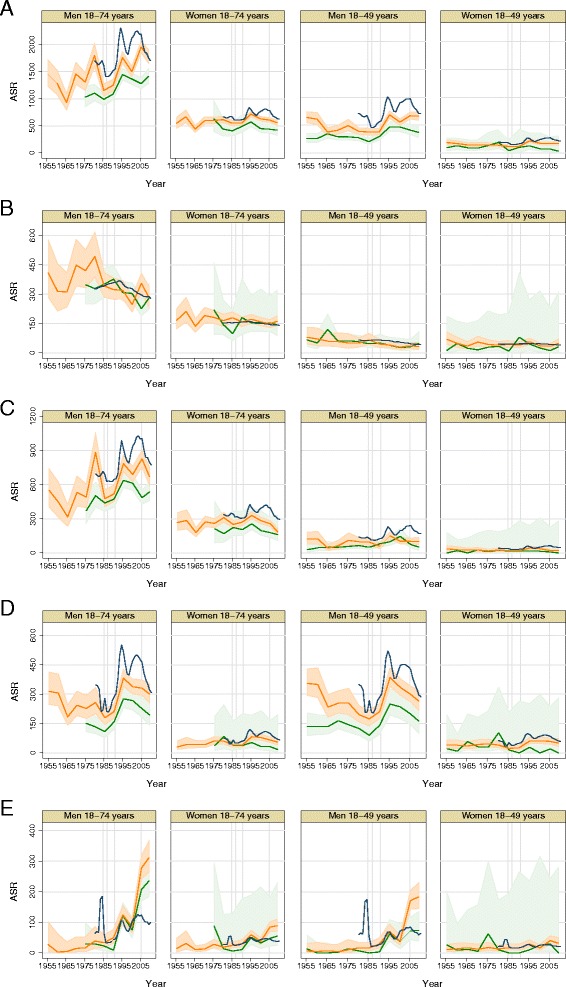
Mortality in the 3-plants workers (green), other Ozyorsk residents (orange) and national figures of Soviet Union and Russia (blue) between 1953 and 2010, by sex for all adults (18–74 years old) and for younger adults (18–49 years old), with indication of years 1985–1987 (anti-alcohol campaign), 1991 (start of political transition), 2005 (start of second anti-alcohol campaign) for **a** All causes of deaths, **b** Malignant neoplasms mortality, **c** Cardiovascular diseases mortality **d** Injury and poisoning **e** Other causes of diseases. Solid line indicates the rate and shaded area the 95 % confidence interval. Abbreviation: ASR: Age standardized rate

Longer term time trends were based on 18,232 deaths in 2,184,608 person-years in the period 1973–2010. They showed similar patterns across the three populations, despite some abrupt fluctuations over time in all cause and non-cancer mortality rates (Table 3, Fig. 1, and Additional file 1: Figure S1). National figures showed the most marked changes which appeared to be attenuated in 3-plants workers (Fig. 1a, c, d). Sixteen pleural cancer deaths were observed (Additional file 1: Figure S1, K).

**Table 3:** Time trends in mortality rates in the population of Ozyorsk city (Russian Federation), 1973–2010

		Men						Women					
		3-Plants workers (352,922 person - years)			Other residents (660,119 person-years)			3-Plants workers (136,804 person-years)			Other residents (1,034,763 person - years)		
Cause of death	ICD-9 codes	ASR	APC	95 % CI	ASR	APC	95 % CI	ASR	APC	95 % CI	ASR	APC	95 % CI
All	001–999	1214	1.0	(0.0, 2.0)	1563	1.1	(−0.3, 2.5)	491	−0.5	(−1.9, 0.9)	603	0.1	(−0.8, 0.9)
All cancers	140–209	316	−1.2	(−2.3, −0.1)	348	−1.0	(−2.4, 0.4)	163	0.2	(−1.6, 2.2)	166	−0.4	(−0.9, 0.0)
Lip, oral cavity, pharynx c.	140–149	9	NA		16	1.2	(−0.7, 3.1)	2	NA		2	NA	
Digestive organs and peritoneum c.	150–159	118	−1.3	(−2.4, −0.2)	139	−1.2	(−2.7, 0.4)	66	−0.9	(−3.2, 1.5)	69	−1.6	(−2.6, −0.7)
*Stomach cancer*	*151*	*46*	*−2.2*	*(−4.1, −0.2)*	*55*	*−2.9*	*(−4.4, −1.2)*	*21*	*−2.2*	*(−7.2, 3.2)*	*24*	*−2.9*	*(−4.3, −1.5)*
*Rectum cancer*	*154*	*21*	*−0.6*	*(−2.6, 1.5)*	*20*	*0.8*	*(−3.0, 4.8)*	*9*	NA		*9*	*−1.5*	*(−3.7, 0.8)*
*Liver cancer*	*155*	*7*	*NA*		*14*	*−5.7*	*(−10.7, −0.3)*	*8*	*−3.3*	*(−8.0, 1.6)*	*4*	*−3.7*	*(−7.4, 0.1)*
*Pancreas cancer*	*157*	*17*	*0.4*	*(−3.0, 3.9)*	*14*	*1.7*	*(−1.4, 5.0)*	*3*	*NA*		*9*	*0.1*	*(−1.8, 2.1)*
Respiratory and intrathoracic c.	160–165	116	−2.4	(−4.2, −0.6)	119	−2.1	(−3.3, −0.8)	18	−2.1	(−7.2, 3.4)	10	−0.4	(−3.3, 2.6)
*Larynx cancer*	*161*	*8*	*3.2*	*(−1.9, 8.4)*	*9*	*NA*		*2*	*NA*		*0*	*NA*	
*Lung cancer*	*162*	*104*	*−2.8*	*(−4.5, −1.1)*	*107*	*−2.7*	*(−3.7, −1.7)*	*15*	*−1.1*	*(−6.3, 4.4)*	*10*	*−0.5*	*(−3.7, 2.9)*
Bone, connective tissues, skin, breast c.	170–175	8	NA		7	2.1	(−2.6, 7.1)	27	3.2	(−0.3, 6.9)	33	1.4	(0.8, 1.9)
*Female breast cancer*	*174*	NA			NA^+^	NA		*21*	*2.2*	*(−1.3, 5.9)*	*29*	*1.6*	*(0.8, 2.5)*
Genitourinary organs c.	179–189	33	−0.5	(−2.9, 2.0)	32	0.2	(−2.0, 2.5)	26	2.8	(−0.3, 6.0)	32	−0.6	(−1.7, 0.6)
Other and unspecified c.	190–199	15	4.5	(1.5, 7.5)	14	0.6	(−1.2, 2.4)	5	NA		10	1.4	(−1.5, 4.5)
Haematological malignancies	200–208	16	−2.6	(−6.7, 1.7)	20	0.1	(−2.9, 3.1)	19	NA		9	0.4	(−2.4, 3.2)
*Multiple myeloma*	*203*	*6*	*NA*		*7*	*NA*		*14*	*NA*		*4*	*NA*	
*Leukemia*	*204*–*208*	*4*	*−3.2*	*(−9.8, 3.9)*	*3*	*1.8*	*(−2.6, 6.5)*	*3*	*NA*		*2*	*NA*	
Infectious and parasitic diseases	001–139	6	NA		11	NA		4	NA		6	NA	
Diseases of circulatory system	390–459	507	0.5	(−1.0, 2.1)	668	0.9	(1.0, 2.7)	206	−1.1	(−2.5, 0.4)	268	−0.5	(−1.9, 0.8)
*Ischemic heart disease*	*410*–*414*	322	*0.4*	*(−1.1, 2.0)*	408	*0.9*	*(−1.0, 2.9)*	99	*−0.3*	*(−3.0, 2.6)*	107	*0.5*	*(−1.1, 2.1)*
*Cerebrovascular disease*	*430*–*438*	129	*0.7*	*(−1.1, 2.5)*	178	*1.3*	*(−0.9, 3.6)*	74	*−2.1*	*(−3.4, −0.7)*	108	*−0.4*	*(−2.3, 1.5)*
Diseases of respiratory system	460–519	39	−1.0	(−3.8, 1.8)	60	−0.7	(−2.5, 1.2)	14	−2.5	(−7.0, 2.2)	15	−3.1	(−5.5, −0.6)
Diseases of digestive system	520–579	59	5.6^a^	(3.9, 7.3)	68	2.8	(0.7, 5.0)	21	2.0	(0.5, 3.4)	31	2.1	(0.9, 3.4)
Diseases of genitourinary system	580–629	7	NA		11	0.4	(−4.3; 5.4)	6	NA		11	−0.3	(−3.7, 3.2)
Injury and poisoning	800–999	189	2.0	(−0.4, 4.5)	278	1.4	(−0.6, 3.5)	41	−1.5	(−4.2, 1.2)	61	0.7	(−1.5, 3.0)
Other causes of death	210–389, 630–799	91	7.4	(2.6, 12.3)	117	8.8	(5.0, 12.7)	36	3.5	(−2.5, 9.8)	47	4.6	(2.1, 7.2)

Legend: Time trends in all cause and cause-specific mortality rates among people aged 18-74 years, workers of the 3 main plant types of Mayak and other Ozyorsk residents (Russian Federation) over period 1973-2010. Abbreviations:  ASR age standardised rate, APC annual percent change, CI confidence interval, c. cancers.

^a^APC for period 1983–2010. Between 1973 and 1983, the APC is −5.6 (−19.1, 10.1)

+1 male breast cancer observed

In men, the all-cause mortality rate increased annually by 1 % among 3-plants workers (95 % CI 0.0, 2.0) and by 1.1 % (95 % CI −0.3 %, 2.5 %) among other residents (Table 3, Fig. 1a). Very marked annual increases were seen in the group of other causes of deaths (7.4 % and 8.8 %) (Fig. 1e), with the figures of the Ozyorsk population appearing to diverge from the national figures since 2003. Increasing rates were also observed in digestive system diseases (annual increases of 5.6 % and 2.8 %) (Additional file 1: Figure S1, V), and in other and unspecified neoplasms in 3-plants workers (4.5 %), while they increased less in other residents (0.6 %) (Additional file 1: Figure S1, O). Circulatory system diseases increased less in 3-plants workers than in other residents (annual increases 0.5 % versus 0.9 %) (Fig. 1c). Rates of cancer deaths decreased annually in 3-plants workers by −1.2 % (95 % CI −2.3 %, −0.1 %) and by −1 % in the other residents (95 % CI −2.4 %, 0.4 %) (Fig. 1b). This overall decrease was driven by decreases in rates of stomach cancer (− 2.2 % in 3-plant workers and −2.9 % in other residents) and lung cancers (−2.8 % in 3-plants workers, and −2.7 % in other residents) (Table 3, Additional file 1: Figure S1 D, I).

In women, the time trends were less strong, but not dissimilar to those in men. Increases were observed for other causes of deaths (annual increases of 3.5 % in 3-plants workers and 4.6 % in other residents), digestive system diseases (2.0 % in 3-plants workers and 2.1 % in other residents), and breast cancers (2.2 % in 3-plants workers and 1.6 % in other residents) (Fig. 1e, Additional file 1: Figure S1 V,M). Genitourinary cancers increased in 3-plants workers (2.8 %) and decreased slightly in other residents (−0.6 %). The overall cancer rates were generally stable, with decreases observed in rates of stomach cancer ( −2.2 % in 3-plant workers and −2.9 % in other residents) and lung cancers (−1.1 % in 3-plants workers and −0.5 % in other residents) (Additional file 1: Figure S1 D, I). Haematological malignancy rate was twice as high among 3-plants workers (ASR =19 based on 20 observed deaths) than among other residents (ASR = 9), but numbers were too small to compare the time trends.

## Discussion

Overall, between 1998 and 2010, the Ozyorsk population showed lower all cause and cause-specific mortality rates than the national figures of the Russian Federation, and 3-plants workers showed even lower mortality rates than the other residents for most causes of deaths. This overall effect is mainly related to lower rates of deaths from circulatory system diseases, and from injury and poisoning. Cancer mortality rates showed smaller differences. Exceptions to this general pattern of lower rates in the Ozyorsk population were seen for the heterogeneous group of other causes of deaths and a few individual cancer sites. Cancer sites which showed elevated rates comparing 3 plants workers and the other Ozyorsk residents were, among men, cancers of other and unspecified sites, and cancers of bone, skin and connective tissue and in women, liver, rectum and lung cancer, multiple myelomas and genitourinary cancers. All these observations were also generally true for the period 1973 to 2010.

Mortality is a result of the frequency of the underlying diseases or events, survival from disease and how well causes of death are registered. One motivation for investigating the mortality of the city of Ozyorsk is the assumed higher exposure to ionising radiation, especially of workers of the nuclear facilities, but perhaps also the other residents due to nuclear accidents in the 1950s [1]. Ionising radiation exposure is known to increase the risk of various cancers [8], and the risk of circulatory system diseases [9]. Three plants workers had a higher average occupational dose than the other residents, although no final documentation of ionising radiation exposure of other residents was available [10]. Dose–response relationships have been observed for lung, liver and bone cancers, for most solid tumours and for cerebrovascular and ischemic heart diseases [4, 5, 11, 12] in Mayak workers. In our analyses at the group level, the ionising radiation exposures did not translate into higher deaths rates for 3 plants workers for most cancers. This was also not the case for death from circulatory diseases. However, some of the cancer sites showing elevated mortality rates in the 3 plants workers had been previously linked to ionising radiation exposure in this cohort (lung and liver cancer in females, bone cancer in men) [11, 13, 14]. Lung, liver and bone were organs with highest plutonium deposition. This likely reflects the long term impact of exposures received in the past, as the induction time of ionising radiation on solid cancers might be long. The interpretation of the other elevated rates was less clear: the high rates of the heterogeneous group of other causes of death and their divergence from the national figures deserves further attention. Death from unknown or unspecified neoplasms could be related to autopsy rate together with differential criteria for recording causes of deaths between 3-plants workers and other residents [3, 14]. The rate of haematological diseases was comparable between 3 plants workers and national figures. However, while the leukaemia rates were decreased, there was an excess of multiple myelomas. They have not often been addressed on their own [11].

Among 40–79 years old, more than 30 % of male and 5 % of female deaths are attributable to tobacco smoking in the Russian Federation between 1980 and 2010 [15]. Alcohol consumption, especially vodka, is a major cause of the high risk of premature death in Russian men [16]. Given the weight of these preventable risk factors, lower prevalences in Ozyorsk and among 3-plants workers especially could explain the lower rates of cardiovascular diseases deaths, of male liver cancers, of digestive system diseases (which includes liver cirrhosis) and of injury and poisoning (including fatal alcohol poisoning and car accidents). We observed that the 7-years decrease in male life expectancy between 1987 and 1994 in Russian Federation [17], and the impact of anti-alcohol campaigns (1985–1987 and since 2005) [18] on cardiovascular mortality and on injury and poisoning deaths decrease in life expectancy was less strong in Ozyorsk and even more in 3-plants workers. This could be due, in part, because 3-plants workers always had a lower alcohol consumption due to their occupation, but no information was available about this for this study.

Higher living standards in Ozyorsk in the 1960’s, 70’s and 80’s in terms of food access and lodging than elsewhere in the Russian Federation/Soviet Union, healthy worker effect whereby persons selected into the workforce are in better general health than the general population, and maintaining a better health through better health surveillance may have contributed to lower deaths rates of the 3-plants workers compared to the other residents. The quality and accessibility of the health care in an organised and affluent city, and for the 3-plants workers specifically, could play a role in the generally lower rates of death compared to Russian Federation, if survival rates were better for the local population than nationally. The very low rate of deaths from infectious diseases, and the low rate of injuries and poisoning deaths could support this explanation, but it could also in part be linked to the isolation of the Ozyorsk population. Affluence might add to the plausibility why some death rates are lower in the Ozyorsk city compared to Russian Federation. Although medical care is free and has been free in the past, cost of medications can be borne by patients, which could influence treatment adherence of chronic conditions (eg hypertension) [19], and cancer survival. Problems of drug supply, even for patients entitled to reimbursement of anti-cancerous treatments have been reported in the context of cancer care in Russian Federation [20]. The higher rates of deaths from multiple myelomas could reflect access to better diagnostic tools for this population compared to the general population, which could impact classification, or differential coding habits. Indeed, the validation of the coding of 246 deaths certificates in the Ozyorsk cause-of-death registry showed that out of the 7 deaths with discrepant coding in the main ICD group, 2 concerned haematological malignancies [3]. The difficulty of the coding of haematological malignancies should not be underestimated.

Finally, there are some uncertainties in the underlying data. Population data is more accurate in census years than between censuses. In addition, the causes of deaths of persons who migrated from the city is unknown. These are limitations especially in the early years where a large part of the workforce was taken from Mayak PA due to the severe working conditions. Deaths among these workers were therefore not recorded in the Ozyorsk cause-of-death registry. Since the 1970’s, migration is very low. Although it cannot be ruled out that some Ozyorsk people with severe disease moved elsewhere, there is no evidence that health per se is a reason for migration.

## Conclusion

Overall, mortality in Ozyorsk was lower than for entire Russia/Soviet Union, suggesting more healthy life in the close city. Mortality patterns of cancers of the most recent years do not suggest a particularly strong radiation effect and only individual-level studies with individual dose estimates are informative with regard to potential radiation-related problems.

## Additional file

Additional file 1: Mortality in the 3-plants workers (green), other Ozyorsk residents (orange) and national figures of Soviet Union and Russian Federation (blue) between 1953 and 2010, by sex for all adults (18-74 years old) and for younger adults (18-49 years old), with indication of year 1985-1987 (anti-alcohol campaign), 1991 (start of political transition), 2005 (start of 2 anti-alcohol campaign) for A - Deaths from infectious and parasitic diseases (ICD-9 codes 001-139), B - Deaths from lip, oral cavity and pharynx cancers (ICD-9 codes 140-149), C - Deaths from digestive organ and peritoneum cancers (ICD-9 codes 150-159), D - Deaths from stomach cancer (ICD-9 code 151), E - Deaths from rectum, rectosigmoid junction and anal cancer (ICD-9 code 154), F - Deaths from liver cancer (ICD-9 code 155), G - Deaths from pancreas cancer (ICD-9 code 157), H - Deaths from respiratory and intrathoracic organ cancers (ICD-9 code 160-165), I- Deaths from trachea, bronchus and lung cancers (ICD-9 code 162), J - Deaths from larynx cancers (ICD-9 code 161), K - Deaths from pleural cancers (ICD-9 code 163), L - Deaths from bone, connective tissue, skin and breast cancers (ICD-9 codes 170-175), M - Deaths from breast cancers, among women (ICD-9 code 174), N - Deaths from genitourinary organs cancers (ICD-9 codes 179-189), O - Deaths from cancers of other and unspecified sites (ICD-9 codes 190-199), P - Deaths from malignant neoplasms of lymphatic and haemopoietic tissue (ICD-9 codes 200-208), Q - Deaths from multiple myelomas (ICD-9 codes 203), R- Deaths from leukemia (ICD-9 codes 204-208), S- Deaths from ischemic heart disease (ICD-9 codes 410-414), T - Deaths from cerebrovascular disease (ICD-9 codes 430-438), U - Deaths from respiratory diseases (ICD-9 codes 460-519), V - Deaths from diseases of the digestive system (ICD-9 codes 520-579), W - Deaths from diseases of the genito-urinary system (ICD-9 codes 580-629). Solid line indicates the rate. Shaded area indicates the 95 % confidence interval for graphs C, S and T. For the other graphs, upper limits of 95 % confidence intervals are truncated to maximum ASR value displayed on scale for graphical reasons (graphs A, D, F, G, H, I, L, M, N O, P, R, U, V, W) or the confidence interval is not shown (graphs B, E, J, K, Q). Abbreviation: ASR: age standardized rate. (DOCX 202 kb)

## References

[1] Kruglov A (2002). The History of the Soviet Atomic Industry.

[2] Koshurnikova NA, Shilnikova NS, Okatenko PV, Kreslov VV, Bolotnikova MG, Sokolnikov ME (1999). Characteristics of the cohort of workers at the Mayak nuclear complex. Radiat Res.

[3] Azizova TV, Fedirko V, Tsareva Y, Tretyakov F, Lassen CF, Friis S (2012). Mayak workers study cohort. An inter-institutional comparison of causes of death in the cause-of-death register of Ozyorsk in the Russian Federation. Methods Inf Med.

[4] Azizova TV, Muirhead CR, Druzhinina MB, Grigoryeva ES, Vlasenko EV, Sumina MV (2010). Cardiovascular diseases in the cohort of workers first employed at Mayak PA in 1948–1958. Radiat Res.

[5] Azizova TV, Haylock RG, Moseeva MB, Bannikova MV, Grigoryeva ES (2014). Cerebrovascular diseases incidence and mortality in an extended Mayak Worker Cohort 1948–1982. Radiat Res.

[6] 6 World Health Organisation: the WHO Mortality Database, http://www.who.int/healthinfo/mortality_data/en/ last accessed feb 2014

[7] Fay MP, Feuer EJ (1997). Confidence intervals for directly standardized rates: a method based on the Gamma distribution. Stat Med..

[8] Kesminiene A, Schüz J. Radiation: ionizing, ultraviolet, and electromagnetic. In World Cancer report. 2014;143–149.

[9] UNSCEAR report on non-cancer effects 2006 http://www.unscear.org/docs/reports/2006/07-82087_Report_Annex_B_2006_Web.pdf last accessed Sept 2015

[10] Koshurnikova NA, Mushkacheva GS, Shilnikova NS, Rabinovich EI, Petrushkina NP, Hall P (2002). Studies on the Ozyorsk population: health effects. Radiat Environ Biophys.

[11] Sokolnikov M, Preston D, Gilbert E, Schonfeld S, Koshurnikova N (2015). Radiation effects on mortality from solid cancers other than lung, liver, and bone cancer in the Mayak worker cohort: 1948–2008. PLoS One.

[12] Azizova TV, Grigorieva ES, Hunter N, Pikulina MV, Moseeva MB (2015). Risk of mortality from circulatory diseases in Mayak workers cohort following occupational radiation exposure. J Radiol Prot.

[13] Sokolnikov ME, Gilbert ES, Preston DL, Ron E, Shilnikova NS, Khokhryakov VV (2008). Lung, liver and bone cancer mortality in Mayak workers. Int J Cancer.

[14] Gilbert ES, Sokolnikov ME, Preston DL, Schonfeld SJ, Schadilov AE, Vasilenko EK (2013). Lung cancer risks from plutonium: an updated analysis of data from the Mayak worker cohort. Radiat Res.

[15] Rentería E, Jha P, Forman D, Soerjomataram I. The impact of cigarette smoking on life expectancy between 1980 and 2010: a global perspective. Tob Control. 2015 Aug 25. doi: 10.1136/tobaccocontrol-2015-052265. [Epub ahead of print] 10.1136/tobaccocontrol-2015-052265 26307052

[16] Zaridze D, Lewington S, Boroda A, Scélo G, Karpov R, Lazarev A (2014). Alcohol and mortality in Russia: prospective observational study of 151,000 adults. Lancet.

[17] Cockerham WC (2000). Health lifestyles in Russia. Soc Sci Med.

[18] Neufeld M, Rehm J (2013). Alcohol consumption and mortality in Russia since 2000: are there any changes following the alcohol policy changes starting in 2006?. Alcohol Alcohol.

[19] Mozheyko M, Eregin S, Vigdorchik A (2012). Hughes D.A cross-sectional survey of hypertension diagnosis and treatment practices among physicians in Yaroslavl Region, Russia. Adv Ther.

[20] Avksentyeva M (2010). Colorectal cancer in Russia. Eur J Health Econ..

