# Supplementary material for: Mortality of populations potentially exposed to ionising radiation, 1953–2010, in the closed city of Ozyorsk, Southern Urals: a descriptive study
Source: Environ Health. 2015 Nov 27;14:91. doi: 10.1186/s12940-015-0078-8 (PMC4661994; doi:10.1186/s12940-015-0078-8)
Supplement: Additional file 1: — Mortality in the 3-plants workers (green), other Ozyorsk residents (orange) and national figures of Soviet Union and Russian Federation (blue) between 1953 and 2010, by sex for all adults (18-74 years old) and for younger adults (18-49 years old), with indication of year 1985-1987 (anti-alcohol campaign), 1991 (start of political transition), 2005 (start of 2 anti-alcohol campaign) for A - Deaths from infectious and parasitic diseases (ICD-9 codes 001-139), B - Deaths from lip, oral cavity and pharynx cancers (ICD-9 codes 140-149), C - Deaths from digestive organ and peritoneum cancers (ICD-9 codes 150-159), D - Deaths from stomach cancer (ICD-9 code 151), E - Deaths from rectum, rectosigmoid junction and anal cancer (ICD-9 code 154), F - Deaths from liver cancer (ICD-9 code 155), G - Deaths from pancreas cancer (ICD-9 code 157), H - Deaths from respiratory and intrathoracic organ cancers (ICD-9 code 160-165), I- Deaths from trachea, bronchus and lung cancers (ICD-9 code 162), J - Deaths from larynx cancers (ICD-9 code 161), K - Deaths from pleural cancers (ICD-9 code 163), L - Deaths from bone, connective tissue, skin and breast cancers (ICD-9 codes 170-175), M - Deaths from breast cancers, among women (ICD-9 code 174), N - Deaths from genitourinary organs cancers (ICD-9 codes 179-189), O - Deaths from cancers of other and unspecified sites (ICD-9 codes 190-199), P - Deaths from malignant neoplasms of lymphatic and haemopoietic tissue (ICD-9 codes 200-208), Q - Deaths from multiple myelomas (ICD-9 codes 203), R- Deaths from leukemia (ICD-9 codes 204-208), S- Deaths from ischemic heart disease (ICD-9 codes 410-414), T - Deaths from cerebrovascular disease (ICD-9 codes 430-438), U - Deaths from respiratory diseases (ICD-9 codes 460-519), V - Deaths from diseases of the digestive system (ICD-9 codes 520-579), W - Deaths from diseases of the genito-urinary system (ICD-9 codes 580-629). Solid line indicates the rate. Shaded area indicates the 95 % confidence interval fo [file 12940_2015_78_MOESM1_ESM.docx]

Additional supporting data for

**Mortality of populations potentially exposed to ionising radiation, 1953-2010, in the closed city of Ozyorsk in the Southern Urals: a descriptive study.**

Deltour I, Tretyakov F, Tsareva Y, Azizova TV and Schüz J.

Table of Contents:

**Figure S1:** Mortality in the 3-plants workers (green), other Ozyorsk residents (orange) and national figures of Soviet Union and Russian Federation (blue) between 1953 and 2010, by sex for all adults (18-74 years old) and for younger adults (18-49 years old), with indication of years 1985-1987 (anti-alcohol campaign), 1991 (start of political transition), 2005 (start of 2^nd^ anti-alcohol campaign) for

- A Deaths from infectious and parasitic diseases (ICD-9 codes 001-139),

- B Deaths from lip, oral cavity and pharynx cancers (ICD-9 codes 140-149),

- C Deaths from digestive organ and peritoneum cancers (ICD-9 codes 150-159),

- D Deaths from stomach cancer (ICD-9 code 151),

- E Deaths from rectum, rectosigmoid junction and anal cancer (ICD-9 code 154),

- F Deaths from liver cancer (ICD-9 code 155),

- G Deaths from pancreas cancer (ICD-9 code 157),

- H Deaths from respiratory and intrathoracic organ cancers (ICD-9 code 160-165),

- I Deaths from trachea, bronchus and lung cancers (ICD-9 code 162),

- J Deaths from larynx cancers (ICD-9 code 161),

- K Deaths from pleural cancers (ICD-9 code 163),

- L Deaths from bone, connective tissue, skin and breast cancers (ICD-9 codes 170-175),

- M Deaths from breast cancers, among women (ICD-9 code 174),

- N Deaths from genitourinary organs cancers (ICD-9 codes 179-189),

- O Deaths from cancers of other and unspecified sites (ICD-9 codes 190-199),

- P Deaths from malignant neoplasms of lymphatic and haemopoietic tissue (ICD-9 codes 200-208),

- Q Deaths from multiple myelomas (ICD-9 codes 203),

- R Deaths from leukemia (ICD-9 codes 204-208),

- S Deaths from ischemic heart disease (ICD-9 codes 410-414),

- T Deaths from cerebrovascular disease (ICD-9 codes 430-438),

- U Deaths from respiratory diseases (ICD-9 codes 460-519),

- V Deaths from diseases of the digestive system (ICD-9 codes 520-579),

-W Deaths from diseases of the genito-urinary system (ICD-9 codes 580-629).

Solid line indicates the rate. Shaded area indicates the 95% confidence interval for graphs C, S and T. For the other graphs, upper limits of 95% confidence intervals are truncated to maximum ASR value displayed on scale for graphical reasons (graphs A, D, F, G, H, I, L, M, N O, P, R, U, V, W) or the confidence interval is not shown (graphs B, E, J, K, Q). Abbreviation: ASR: age standardized rate.
